# Soil microbial community variation correlates most strongly with plant species identity, followed by soil chemistry, spatial location and plant genus

**DOI:** 10.1093/aobpla/plv030

**Published:** 2015-03-27

**Authors:** Jean H. Burns, Brian L. Anacker, Sharon Y. Strauss, David J. Burke

**Affiliations:** 1 Department of Biology, Case Western Reserve University, Cleveland, OH 44106, USA; 2 University of California, Davis, CA, USA; 3 The Holden Arboretum, 9500 Sperry Road, Kirtland, OH 44094, USA

**Keywords:** Coastal grassland community, niche, soil bacterial community, soil fungal community, terminal-restriction fragment length polymorphism

## Abstract

Soil ecologists have debated the relative importance of dispersal limitation and ecological factors in determining the structure of soil microbial communities. Recent evidence suggests that ‘everything is not everywhere’, and that microbial communities are influenced by both dispersal limitation and ecological factors. However, we still do not understand the relative explanatory power of spatial and ecological factors, including plant species identity and even plant relatedness, for different fractions of the soil microbial community (i.e. bacterial and fungal communities). To ask whether factors such as plant species, soil chemistry, spatial location and plant relatedness influence rhizosphere community composition, we examined field-collected rhizosphere soil of seven congener pairs that occur at Bodega Bay Marine Reserve, CA, USA. We characterized differences in bacterial and fungal communities using terminal-restriction fragment length polymorphism. Plant species identity was the single best statistical predictor of both bacterial and fungal community composition in the root zone. Soil microbial community structure was also correlated with soil chemistry. The third best predictor of bacterial and fungal communities was spatial location, confirming that everything is not everywhere. Variation in microbial community composition was also related to combinations of spatial location, soil chemistry and plant relatedness, suggesting that these factors do not act independently. Plant relatedness explained less of the variation than plant species, soil chemistry, or spatial location. Despite some congeners occupying different habitats and being spatially distant, rhizosphere fungal communities of plant congeners were more similar than expected by chance. Bacterial communities from the same samples were only weakly similar between plant congeners. Thus, plant relatedness might influence soil fungal, more than soil bacterial, community composition.

## Abstract (precis)

Soil ecologists have debated the relative importance of dispersal limitation ("everything" is not "everywhere") and ecological factors in determining the structure of soil microbial communities. The relative explanatory power of spatial and ecological factors, including plant species identity and even plant relatedness, for different fractions of the soil microbial community (i.e. bacterial and fungal communities) are poorly understood. We sampled field soils in a northern California field site, and find that soil microbial community variation correlates most strongly with plant species identity, followed by soil chemistry, spatial location and plant genus.

## Introduction

The study of microbial community structure has long centred around the debate between the hypothesis that ‘everything is everywhere’ (Bass Becking 1934), and the alternative that dispersal limitation influences microbial community structure (O'Malley 2007). Because the soil microbial community is diverse, heterogeneous and difficult to characterize (Singh *et al.* 2004), understanding the environmental correlates of soil microbial communities has lagged behind community ecology studies in other systems (Fierer and Ladau 2012; Fierer *et al.* 2012). Thus the relative explanatory powers of ecological factors (e.g. plant species identity, soil chemistry and plant relatedness) and dispersal limitation (i.e. spatial location) for determining soil microbial communities are not well characterized (reviewed in Berg and Smalla 2009). By comparing bacterial and fungal community datasets, we explore whether ecological and spatial factors structure soil microbial communities, and, if so, how bacterial and fungal communities differ.

Plant–microbial relationships are often plant species-specific (reviews in Ehrenfeld *et al.* 2005; Hardoim *et al.* 2008; Berg and Smalla 2009). For example, legume species are often associated with particular strains or species of rhizobia bacteria (Long 2001). Plants exude chemicals from their roots that can foster beneficial microbes in the rhizosphere (reviewed in Ehrenfeld *et al.* 2005; Bais *et al.* 2006; Compant *et al.* 2010). For example, *Arabidopsis thaliana* exudes malic acid in the presence of a pathogen, and malic acid attracts a beneficial bacterium, *Bacillus subtilis*, protecting the roots from the pathogen (Rudrappa *et al.* 2008). However, plant species-specific relationships are not universally found for soil microbial communities (Ehrenfeld *et al.* 2005), and data sets comparing bacterial and fungal communities for plant species-specificity are rare (but see, e.g. Ushio *et al.* 2008).

Soil chemistry also influences soil microbial community composition, diversity and activity (Ehrenfeld *et al.* 2005). For example, differences in pH explained 70 % of the variance in bacterial community diversity across soils sampled from North and South America (Fierer and Jackson 2006). Greater soil fertility (NPK) increases bacterial biomass and activity in grassland mesocosms and these effects interact with plant species identity in some systems (Innes *et al.* 2004) and not in others (Bardgett *et al.* 1999), thus effects of soil chemistry are often system-dependent. Whether or how these soil chemistry effects may interact with plant species identity or plant relatedness is not generally known.

Spatial location could also influence soil microbial composition, especially if microbial taxa are dispersal limited, resulting in spatial autocorrelation of microbial communities (e.g. Ettema and Wardle 2002; Fierer and Ladau 2012). Early workers argued that microbes do not experience significant dispersal limitation, and thus ‘everything is everywhere: but the environment selects’ (Bass Becking 1934, reviewed in O'Malley 2007). However, more recent work has found strong spatial autocorrelation in many microbial communities, such that spatially close communities are more similar than expected by chance, and this similarity decays with distance (Ettema and Wardle 2002). Such a pattern of spatial autocorrelation suggests that dispersal limitation does influence patterns of distribution for microbes (O'Malley 2007; Rout and Callaway 2012). The relative importance of dispersal limitation and ecological factors is still an area of ongoing research (Berg and Smalla 2009).

If closely related plants are similar in root morphology (e.g. Comas and Eissenstat 2009; Valverde-Barrantes *et al.* 2014), root exudates, or other drivers of microbial associations, plant relatedness might also be expected to correlate with soil microbial community structure. Consistent with this hypothesis, plant clades, in some cases, have similar responses to soil biota (e.g. Brandt *et al.* 2009; Liu *et al.* 2012; Reinhart *et al.* 2012; Anacker *et al.* 2014). Closely related plants also often share characteristics of root morphology, such as the absence of many fine roots (Valverde-Barrantes *et al.* 2014), a trait which is correlated with higher mycorrhizal colonization (Manjunath and Habte 1991; Brundrett 2002). In addition, there is evidence, especially in Orchidaceae, of closely related plants sharing similar soil fungal associates (Jacquemyn *et al.* 2011; Martos *et al.* 2012) and close relatives sometimes respond similarly to arbuscular mycorrhizal fungi (e.g. Reinhart *et al.* 2012; Lugo *et al.* 2015). However, such relationships are by no means universal, and there are cases were plant relatedness does not correlate with effects of soil biota (e.g. Reinhart and Anacker 2014). Previous studies have often focused on whole-soil effects (i.e. plant-soil feedbacks, e.g. Burns and Strauss 2011) or have focused on a narrow fraction of the soil community (e.g. mycorrhizae, Reinhart *et al.* 2012; Reinhart and Anacker 2014); therefore, there is little comparative evidence about the relative influence of plant relatedness on different components of the soil microbiome (e.g. bacterial and fungal communities).

We quantified the relative contributions of plant species, soil chemistry, spatial location and plant relatedness on soil microbial communities at Bodega Bay Marine Reserve, CA, USA. (i) We predicted that plant species identity may influence soil microbial community composition, if species differ in factors, such as root exudates, which influence microbial community composition. (ii) Soil chemistry (e.g. N, pH) may correlate with microbial communities, for example, if soil microbe species differ in their nutrient preferences or soil chemistry niche axes. (iii) Spatial separation may influence microbial community composition across samples, if microbes are dispersal limited. (iv) Finally, plant congeners may have similar soil microbial communities, if close relatives have similar traits or habitat preferences, which influence soil microbial community composition.

## Methods

To explore microbial community composition, we sampled field soils and used DNA fragment analysis to characterize differences among soil samples. For each of the 14 angiosperm species, we sampled soils from 6 unique collection locations in the field, at least 100 m apart, at Bodega Bay Marine Reserve, CA, USA [**see Supporting Information**]. These species are *Cirsium occidentale*, *C. quercetorum*, *Fragaria chiloensis*, *F. vesca*, *Gilia capitata* ssp. *chamissonis*, *G. millefoliata*, *Plantago erecta*, *P. subnuda*, *Rumex crassus*, *R. occidentalis*, *Sanicula arctopoides*, *S. crassicaulis*, *Trifolium fucatum* and *T. gracilentum*. Soil samples were collected from the root zone of each focal plant species to 15 cm depth, roots were removed and soils were mixed within a core. Soils therefore represent rhizosphere and non-rhizosphere soil within the active root zone of the selected plants species, which we expect to be under the influence of the plant and therefore represent microbial communities cultivated by the influence of the representative plant species. Soil samples were stored on dry ice in the field and moved to −80 °C within <12 h. To quantify the role of spatial proximity, we mapped collection locations using GPS coordinates.

### Soil bacterial and fungal communities

Terminal-restriction fragment length polymorphism (TRFLP) was used to characterize the soil bacterial and fungal communities following established methods (as in, for example, Burke *et al.* 2008). Terminal-restriction fragment length polymorphism is a DNA fragmentation technique that quantifies differences in the soil microbial community among samples but which cannot identify microbes to specific taxa. Terminal-restriction fragment length polymorphism estimates of microbial community structure have been found to be generally robust and concur with alternative approaches such as 454 pyrosequencing in documenting microbial community differences (Pilloni *et al.* 2012). See details of the TRFLP methodology in the **Supporting Information**. In brief, for each soil sample, DNA was extracted from two 500-mg subsamples of collected soil, amplified using PCR to target bacterial or fungal-specific target regions and restriction enzymes were used to digest the PCR product. We then averaged the subsample extractions within a soil sample for analysis. Terminal-restriction fragment length polymorphism results in size fragments that represent operational taxonomic units (OTUs) in our analyses.

### Soil chemistry

To characterize soil chemical properties, soil samples taken from each soil collection (*n* = 84) were analysed for NO_3_ (ppm), Olsen P (ppm), K (ppm), K (meq/100 g), Na (ppm), Ca (meq/100 g), Mg (meq/100 g), cation-exchange capacity, organic matter and pH by the Division of Agriculture and Natural Resources Analytical Laboratory at the University of California, Davis.

### Phylogeny estimation

We sampled seven congeneric pairs and six soil replicates per species, and two subsamples per soil sample, to assess heterogeneity in soil microbial communities. We tested for effects of plant phylogeny, as well as tested just for effects of plant genus on microbial community composition (see below).

To estimate the phylogeny among our sampled plant species, we downloaded all available DNA regions for *mat*K, ITS and *trn*L-*trn*F from genBank for the 14 sampled plant species **[see Supporting Information]**. Each species was represented in the data set by at least 1 DNA region, *mat*K was present for 7 species, ITS for 13 and *trn*L-*trn*F for 11 [**see Supporting Information**]. We aligned the sequences separately for each DNA region with MEGA version 5.2.2 (Tamura *et al.* 2011) using MUSCLE (Edgar 2004*a*, *b*) with the default gap open penalty of −400 and then concatenated the alignments. We conducted a maximum likelihood search using Garli version 0.951 (Zwickl 2006) using a GTR + Γ + I model of evolution. To incorporate prior knowledge about angiosperm phylogeny, we enforced a constraint tree with the known relationships among genera within the Asterids (Stevens 2009). Bootstrap analysis with 100 replicates was conducted without constraints. Thus bootstrap values reflect repeatability of clades, based on the raw DNA data. We calibrated the branch lengths using r8s with the NPRS method (Sanderson 2003) and dates fixed at the Fabaceae, Rosaceae, Asteraceae, Apiaceae, Polygonaceae, Lamiales, Caryophyllales, coreeudicots, eurosid1 and easterid2 based on published fossil calibration dates (Wikström *et al.* 2001).

### Data analysis

#### Soil community diversity

We ordinated the community data using non-metric multidimensional scaling. We used the ‘metaMDS’ function in the ‘vegan’ package (Oksanen *et al.* 2010), using 20 random starts and a Bray–Curtis distance matrix (Faith *et al.* 1987). We conducted ordinations with and without data transformation, and with and without removing rare taxa (taxa present in ≤7 % of subsamples) prior to ordination. These strategies did not reduce ordination stress, and we therefore present ordinations on untransformed whole community data. In addition, we conducted visual inspection of a graph of stress versus the number of dimensions in the ordination, and chose the number of axes that reduced stress below 0.20, where the graph appeared to asymptote (*k* = 4). NMDS ordination resulted in a reduced number of dimensions that describe differences in soil communities, and with which we could conduct further analyses.

To describe the amount of community variance potentially represented by the ordination, we conducted a post hoc partial Mantel correlation between a Euclidean distance matrix from the ordination and the total community data matrix using Bray–Curtis distances following recommendations in McCune and Grace (2002). We used *multi.mantel* in the phytools package with 1000 permutations (Revell 2012) to conduct this analysis and present *R*^2^ values converted to percentages.

#### Do plant species identity, soil chemistry, spatial location and phylogenetic relatedness predict similarity in rhizosphere microbial communities?

We used two statistical tools to explore the correlations in this data set: phylogenetic eigenvector regression (PVR) and a variance partitioning analysis. The PVR approach allows us to test for statistical significance of effects of our predictors on soil microbial community structure. Because many predictors may covary in this observational data set, the variance partitioning approach allows us to ask about the relative explanatory power of combinations of predictors and is purely descriptive.

First, we fit four PVR models, each with one of the ordination axes as the response variable; predictors in each model included the eigenvectors that represented plant species, soil chemistry, spatial location and plant phylogeny, as described below. To acquire phylogenetic eigenvectors, we first decomposed the phylogeny with 14 plant taxa using *PVRdecomp* in the PVR library (Diniz-Filho *et al.* 1998, 2012). We examined a graph of the eigenvalues against the vector number for the decomposition and used a visual break in the data to determine which eigenvectors to consider for the model selection, which included the first seven. Next, we decomposed spatial distance into a set of two eigenvectors (Bellier *et al.* 2007). To select a minimal model for each ordination axis, we fit a full linear model with the first seven phylogenetic eigenvectors and two spatial eigenvectors using the *lm* function. We then choose the subset of phylogenetic eigenvectors that were statistically significant (*P* < 0.05) for the reduced model (Diniz-Filho *et al.* 2012). This approach never resulted in more than three phylogenetic predictors. Because identical values for eigenvectors were associated with each species, species identity could not simultaneously be included in this model. We compared this full model (e.g. fungal community axis 1 ∼ plant phylogeny + spatial location) to models dropping effects from the model using a likelihood ratio test to determine the statistical significance of each predictor. Because ‘phylogeny’ and ‘spatial location’ consisted of multiple vectors, this approach tested the overall significance of these predictors of microbial community structure. To select minimal soil chemical models, we used stepAIC on a model with all soil chemical vectors and included only statistically significant predictors in the final model. This reduced variance inflation factors to below 2.5 for all predictors. Because phylogeny co-varied with soil chemistry (Spearman rank correlation range: −0.44 to 0.59) and models with both phylogeny and soil chemistry resulted in high multicollinearity (VIF > 100), phylogeny and soil chemistry effects were tested separately. Finally, we report the adjusted *R*^2^ for models with each of four classes of predictors (i.e. plant species, soil chemistry, spatial location and plant phylogeny) individually, to estimate the maximum relative explanatory power of each, adjusted for the number of predictors in the model. Because our sampling was concentrated on congeners, we also conducted a PVR analysis with plant genus as a predictor, rather than plant phylogeny, to ask whether plant genus alone could predict differences in microbial community composition.

Standard diagnostic plots were examined and residuals were normally distributed for all PVR models. We used adjusted *R*^2^ value comparisons among models to ensure that models were not over-fit. Variance inflation factors were <2.5 for all predictors in all final models (median VIF = 1.03), which is generally considered very low multicollinearity (Fox and Monette 1992; O'Brien 2007).

Finally, some of these predictors may covary, such as if closely related species prefer the same habitats and are thus spatially close together, and multiple predictors combined could influence microbial community composition. To describe the combined effects of plant phylogeny, soil chemistry and spatial location on soil community structure, we used variance partitioning methods (Desdevises *et al.* 2003; Peres-Neto *et al.* 2006; Gonçalves-Souza *et al.* 2014). See the **Supporting Information** for more details about this analysis. All analyses were conducted in the R statistical package (version 3.0.2) (R Development Core Team 2008). Data are available from the Dryad Digital Repository: http://dx.doi.org/10.5061/dryad.9bf7f.

## Results

### Soil chemistry

Soils from the Bodega Bay site were primarily loamy sand with a somewhat acidic average pH of 6.07 ± 0.05 SE (range = 5.01–8.03). Organic matter was generally low with a mean 4.2 % ± 0.2 SE (range = 0.6–15.5 %). Salinity was highly variable and ranged from 12 to 390 ppm with a mean of 91.8 ± 6.7 SE at this coastal site.

### Bacterial communities

**Figure 1.**
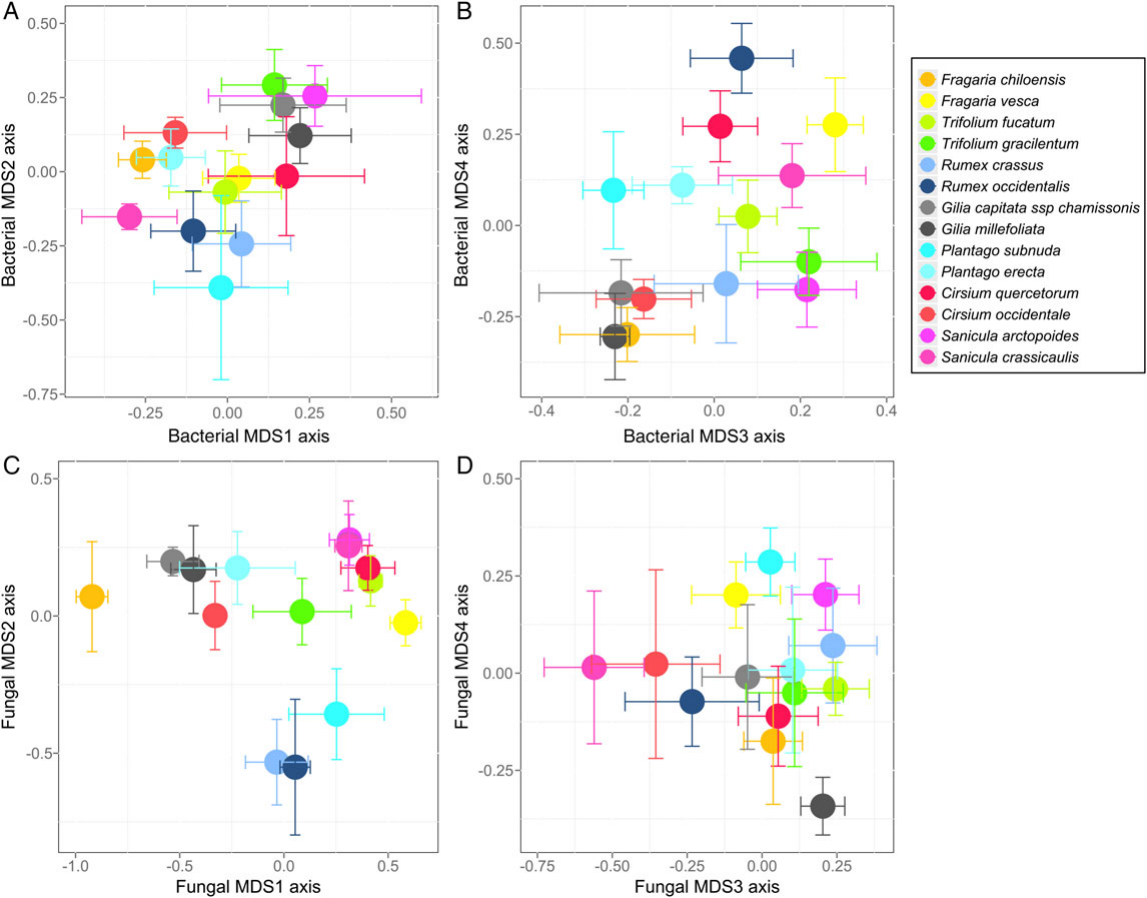
NMDS ordination with species means ± 1 SE for soil samples collected in the field at Bodega Bay, CA from the rhizospheres of 14 plant species. (A and B) The bacterial community ordination with a stress of 0.12. (C and D) The fungal community ordination with a stress of 0.15.

**Figure 2.**
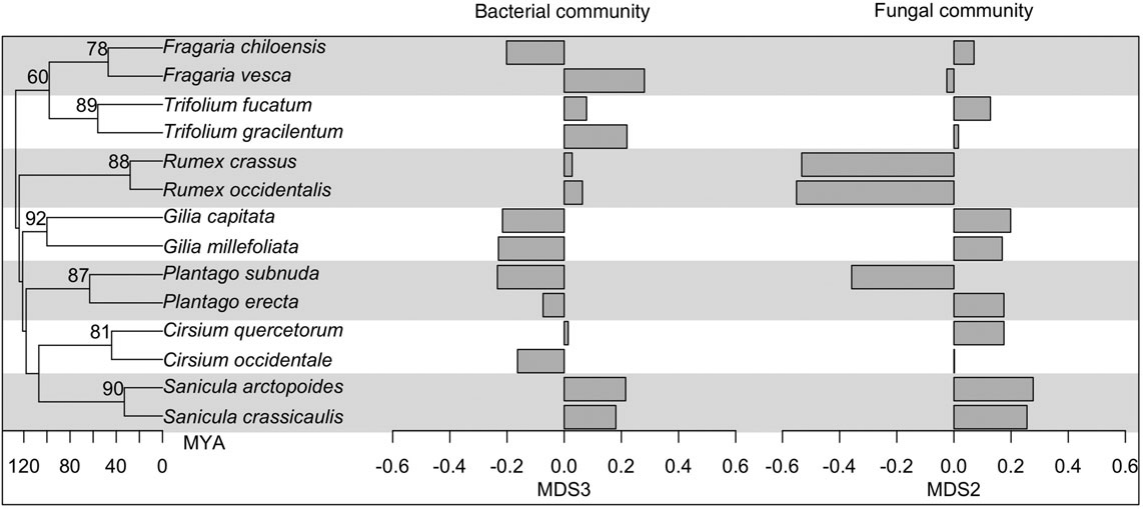
Soil bacterial communities were significantly, but weakly, correlated with plant phylogeny on ordination axis 3 (Table 1). Plant phylogeny explained 29 % of the variance in soil fungal communities on ordination axis 2 (Table 1).

NMDS ordination resulted in a stress of 0.12 with four ordination axes (Fig. 1A and B), and the total ordination (axes 1–4 combined) explained ∼82 % of the bacterial community composition in a partial Mantel test. Bacterial communities in the rhizosphere exhibited plant species-specific patterns, and plant species explained 15, 15 and 38 % of the variance on ordination axes 2, 3 and 4, respectively (Table 1). Variation in pH explained 19 % of the variance in bacterial community composition on axis 2 (Table 1). Variation in Mg and Ca across samples explained 16 % of the variation on axis 3 (Table 1). Variation in K, Na and Ca explained 11 % of the variance on axis 4 (Table 1). Spatial location explained 19 and 16 % of the variation on ordination axes 3 and 4 (Table 1). Plant phylogeny was a significant but poor predictor of bacterial community composition, explaining 1, 5, 9 and 8 % of the variation on ordination axes 1–4 (Table 1). An alternative analysis approach with plant genus instead of plant phylogeny explained 12 % of the variance on ordination axis 3 **[see Supporting Information—Table S2]**, and this pattern was primarily driven by the *Gillia* and *Sanicula* congeners, which were very similar in their bacterial community composition on axis 3 (Fig. 2). In the variance partitioning analysis, soil chemistry alone explained more of the variance in bacterial community composition than other factors, and combinations of predictors (e.g. spatial location, soil chemistry and plant phylogeny combined), explained additional variation **[see Supporting Information—Fig. S2]**.

**Table 1.** Linear PVR models were used to test the effects of plant species, soil chemistry, spatial location and plant relatedness on soil microbial community composition. Model selection was conducted by retaining statistically significant phylogenetic eigenvectors (*P* < 0.05) for the combined data set (see text for details). Significant results (*P* < 0.05) are highlighted in bold.

Predictor	Microbial community structure ordination axes							
	MDS1		MDS2		MDS3		MDS4	
	Adj. *R*^2^	*P*-value	Adj. *R*^2^	*P*-value	Adj. *R*^2^	*P*-value	Adj. *R*^2^	*P*-value
Bacterial								
Plant species	<0.01	0.42	**0**.**15**	**0**.**02**	**0**.**15**	**0**.**02**	**0**.**38**	**<0**.**001**
Soil chemistry	<0.01	0.77	**0**.**19**	**<0**.**001**	**0**.**16**	**<0**.**01**	**0**.**11**	**0**.**05**
Spatial location	<0.01	0.10	<0.01	0.50	**0**.**19**	**<0**.**001**	**0**.**16**	**<0**.**01**
Phylogeny	**0**.**01**	**0**.**02**	**0**.**05**	**0**.**02**	**0**.**09**	**0**.**02**	**0**.**08**	**0**.**02**
Fungi								
Plant species	**0**.**56**	**<0**.**001**	**0**.**31**	**<0**.**001**	**0**.**20**	**<0**.**01**	0.03	0.31
Soil chemistry	**0**.**45**	**<0**.**001**	**0**.**27**	**<0**.**001**	<0.01	0.17	0.03	0.06
Spatial location	**0**.**43**	**<0**.**001**	<0.01	0.45	**0**.**09**	**<0**.**001**	**0**.**10**	**0**.**05**
Phylogeny	<0.01	0.07	**0**.**29**	**<0**.**001**	**<0**.**01**	**<0**.**01**	0.02	0.66

### Fungal communities

The NMDS ordination for the fungal data set had a stress of 0.15 (Fig. 1C and D), and the total ordination (axes 1–4 combined) explained ∼71 % of the fungal community composition in a partial Mantel test. Plant species identity explained 56, 31 and 20 % of the variation on ordination axes 1, 2 and 3, respectively (Table 1). Phosphorus, Ca and Mg explained 45 % of the variance on fungal axis 1 (Table 1). Variation among soil samples in pH explained 27 % of the variation in fungal community composition on axis 2 (Table 1). Spatial location also explained a significant amount of the variance in fungal community composition, explaining 43, 9 and 10 % of the variance on ordination axes 1, 3 and 4 (Table 1). Plant phylogeny explained 29 % of the variance on ordination axis 2 (Table 1). The alternative analysis with plant genus, rather than plant phylogeny, suggested that plant genus explained 16 and 30 % of the variance on ordination axes 1 and 2, respectively **[see Supporting Information—Table S2]**. For example, *Rumex* species fungal rhizosphere communities were very close in ordination space (Fig. 1C, blue points) as were *Gillia* congeners (Fig. 1C, grey points), and *Sanicula* congeners (Fig. 1C, pink points). This signal of plant genus was especially strong on ordination axis 2 (Fig. 2, **Supporting Information—Table S2**). Combinations of the predictors explained larger percentages of the fungal community composition than individual predictors alone **[see Supporting Information—Fig. S2]**.

## Discussion

### Plant species identity was the single best statistical predictor of soil microbial community composition

Consistent with a large body of literature (reviews in Brundrett 2002; Ehrenfeld *et al.* 2005; Berg and Smalla 2009), we found a signal of plant species identity on the soil bacterial and fungal communities, and plant species was the single best statistical predictor of soil microbial community variation (Table 1). Variance in bacterial community composition was higher than for fungal communities, and no combination of predictors could explain a large amount of this variance **[see Supporting Information—Fig. S2]**. This large variance could reflect a high degree of fine-scale heterogeneity within the root zone to which bacterial communities respond. For example, the presence of fungal biomass was found to influence the community structure of important bacterial functional groups (e.g. denitrifying bacterial) at fine spatial scales within a forest system (Burke *et al.* 2012). This high heterogeneity of microsites for bacterial within the root zone, coupled with the high degree of species diversity within the bacterial domain, could be responsible for the weaker predictors of bacterial communities in our study. In addition, our examination of bacterial community structure using general 16S rDNA primers could miss the potential relationship between plant species and specific bacterial functional groups. Analysis of the nitrogen-fixing functional group, for example, could reveal plant effects on that functional group, because we expect some plants to have co-evolved relationships with these nitrogen-fixing bacteria (e.g. rhizobia-plant relationships; Long 2001). The weaker signal of plant species identity on bacteria could therefore be due to our focus on overall bacterial community structure and not functional group distribution, which could yield different results.

Plant species explained a significant amount of the variance in fungal community composition (Table 1). Effects of plant species identity on soil microbial communities might be the result of ‘active’ selection by plants for soil microbes (e.g. via root exudates), a ‘passive’ by-product of nutrient uptake, root structure (Singh *et al.* 2004) or shared habitat preferences by plants and microbes. It is increasingly accepted that soil fungi, and especially mutualistic organisms such as mycorrhizal fungi (Brundrett 2002), may form plant species-specific relationships that can affect plant growth and community composition. Plant species-specific associations with soil microbes likely contribute to the strong plant-soil feedbacks observed in many systems (Kulmatiski *et al.* 2008), which have important implications for coexistence and community assembly (Bever *et al.* 2010).

### Soil chemistry was correlated with soil microbial community composition

Soil chemistry had the second strongest correlation with differences in microbial communities (Table 1). Previous studies have found strong correlations between soil chemistry and soil microbial communities at large spatial scales (e.g. Lauber *et al.* 2009), consistent with our results at smaller spatial scales. Variation in pH is often correlated with bacterial communities at the continent scale (Lauber *et al.* 2009), and we found pH to correlate with bacterial community composition. Fungal communities, including AMF and EMF communities, also frequently differ across soils with differences in pH (Kluber *et al.* 2012), also consistent with the correlation between fungal ordination axis 2 and pH. Further, many fungi can affect C and N availability and cycling through their saprotrophic capabilities (e.g. production of extracellular enzymes for liberation of organic nutrients) (Leake *et al.* 2002), though we found no strong evidence for correlations between the fungal community and N.

### Spatial location was the third best predictor of microbial community composition and was correlated with soil chemistry and plant relatedness

We found significant associations between soil microbial communities and spatial location (Table 1). Thus ‘everything’ is not ‘everywhere’ in this data set, as many other workers have also found for soil microbial communities (reviews in Ettema and Wardle 2002; Rout and Callaway 2012). This is consistent with the hypothesis that dispersal limitation influences microbial distributions even at fairly restricted spatial scales **[see Supporting Information—Fig. S1]**. The correlations we observed between soil chemistry, plant relatedness and spatial locations on fungal community composition suggest that these factors co-varied in the field, making disentangling the relative importance of these predictors difficult **[see Supporting Information—Fig. S2]**.

### Plant congeners had similar soil fungal, but not bacterial, communities

Plant relatedness explained less of the variation in soil microbial communities than plant species, soil chemistry or spatial location (Table 1). Plant congeners had similar soil fungal (but not bacterial) communities, even when they occurred in divergent habitats. For example, congeners in *Sanicula* and *Rumex* had similar fungal communities (Fig. 2) but they are found in different habitats and are spatially distant (>1 km) from one another **[see Supporting Information—Fig. S1]**. Thus, spatial location and similarity in habitat preferences between close relatives are not the sole drivers of the correlation between plant relatedness and soil fungal communities. Similar root exudates or root morphology between close relatives (Comas and Eissenstat 2009; Valverde-Barrantes *et al.* 2014) may be other explanations for shared fungal community composition.

DNA fragmentation tools, like TRFLP, do not identify taxa without additional work. Because our soil sampling procedure may have broken some plant roots, endophytic fungi could be contributing to the pattern we observed. Our general ITS fungal primers preferentially amplify non-arbuscular mycorrhizal fungi (D. J. Burke, pers. obs.; Taylor *et al.* 2014), suggesting that arbuscular mycorrhizal fungi are not driving the correlation that we observed between plant phylogeny and fungal rhizosphere community. Given the species-specific nature of some mycorrhizal associations (Brundrett 2002), we may be underestimating the importance of host plant relatedness in influencing fungal rhizosphere communities.

Meta-analyses suggest that plant relatedness does not explain much of the overall variance in plant-soil feedbacks, where plant performance is compared between conspecific soils and heterospecific soils (Mehrabi and Tuck 2015); however, phylogenetic relatedness in plant-soil feedbacks did improve predictions of plant abundance for 57 species within an old field community (Anacker *et al.* 2014). Some fractions of the soil community may be similar between closely related plants. For example, arbuscular mycorrhizal effects are similar between closely related plants (Reinhart *et al.* 2012; Lugo *et al.* 2015) and some mycorrhizal associates are specialized to particular plant clades (Brundrett 2002). Our results suggest that plant relatedness correlates with the fungal fraction of the soil community, but only weakly with the bacterial community. Thus the relative importance of fungal and bacterial components of the soil microbiota to plant performance may influence whether close relatives have similar plant–soil feedback effects on one another (Burns and Strauss 2011).

## Conclusions

Plant species identity was the best predictor of bacterial and fungal community composition in the soil, followed by soil chemistry, spatial location and plant relatedness. Because ‘everything’ is not ‘everywhere’ in microbial communities, spatial location can confound our attempts to predict microbial community structure. Future work should use experimental manipulations to disentangle covarying predictors, such as soil chemistry, spatial location and plant relatedness in the field. Understanding the feedbacks between soil microbial community structure and plant communities may benefit from considering the evolutionary relationships among plants.

## Sources of Funding

Our work was funded by the National Science Foundation (United States) DEB (11-20387) and by the Holden Arboretum Trust and the Corning Institution for Education and Research.

## Contributions by the Authors

J.H.B. contributed to data collection and analysis and wrote the manuscript. B.L.A. collected soil samples in the field, sent soil samples for nutrient analysis and contributed to data analysis. S.Y.S. co-conceived the project and data collection design, discussed data analysis and commented on drafts. D.J.B. trained J.H.B. and undergraduate researchers in TRFLP techniques in the laboratory, mentored undergraduates in the laboratory and contributed to data analysis and manuscript writing. All authors contributed to manuscript revision.

## Conflict of Interest Statement

None declared.

## Supporting Information

The following additional information is available in the online version of this article –

**Table S1.** Plant species sampled to examine rhizosphere soil microbial communities at Bodega Bay, CA, USA.

**Figure S1.** Map of the spatial sampling locations for the 14 species and 6 replicate soil collections per species at Bodega Bay, CA, USA.

**Figure S2.** Variance partitioning analysis on the bacterial and fungal data sets.

**Table S2.** Linear PVR models were used to test the effects of plant species, spatial location and plant genus on soil microbial community composition.

Additional Information

## References

[PLV030C1] Anacker BL Klironomos JN Maherali H Reinhart KO Strauss SY 2014 Phylogenetic conservatism in plant-soil feedback and its implications for plant abundance. Ecology Letters 17:1613–1621. 10.1111/ele.12378 25328022

[PLV030C2] Bais HP Weir TL Perry LG Gilroy S Vivanco JM 2006 The role of root exudates in rhizosphere interactions with plants and other organisms. Annual Review of Plant Biology 57:233–266. 10.1146/annurev.arplant.57.032905.105159 16669762

[PLV030C3] Bardgett RD Mawdsley JL Edwards S Hobbs PJ Rodwell JS Davies WJ 1999 Plant species and nitrogen effects on soil biological properties of temperate upland grasslands. Functional Ecology 13:650–660. 10.1046/j.1365-2435.1999.00362.x

[PLV030C4] Bass Becking L 1934 Geobiologie of Inleiding Tot de Milieukunde. The Hague: Van Stockum & Zoon.

[PLV030C5] Bellier E Monestiez P Durbec J-P Candau J-N 2007 Identifying spatial relationships at multiple scales: principal coordinates of neighbour matrices (PCNM) and geostatistical approaches. Ecography 30:385–399. 10.1111/j.0906-7590.2007.04911.x

[PLV030C6] Berg G Smalla K 2009 Plant species and soil type cooperatively shape the structure and function of microbial communities in the rhizosphere. FEMS Microbiology Ecology 68:1–13. 10.1111/j.1574-6941.2009.00654.x 19243436

[PLV030C7] Bever JD Dickie IA Facelli E Facelli JM Klironomos J Moora M Rillig MC Stock WD Tibbett M Zobel M 2010 Rooting theories of plant community ecology in microbial interactions. Trends in Ecology and Evolution 25:468–478. 10.1016/j.tree.2010.05.004 20557974 PMC2921684

[PLV030C8] Brandt AJ Seabloom EW Hosseini PR 2009 Phylogeny and provenance affect plant–soil feedbacks in invaded California grasslands. Ecology 90:1063–1072. 10.1890/08-0054.1 19449700

[PLV030C9] Brundrett MC 2002 Coevolution of roots and mycorrhizas of land plants. New Phytologist 154:275–304. 10.1046/j.1469-8137.2002.00397.x 33873429

[PLV030C10] Burke DJ Dunham SM Kretzer AM 2008 Molecular analysis of bacterial communities associated with the roots of Douglas fir (*Pseudotsuga menziesii*) colonized by different ectomycorrhizal fungi. FEMS Microbiology Ecology 65:299–309. 10.1111/j.1574-6941.2008.00491.x 18459969

[PLV030C11] Burke DJ Smemo KA López-Gutiérrez JC DeForest JL 2012 Soil fungi influence the distribution of microbial functional groups that mediate forest greenhouse gas emissions. Soil Biology and Biochemistry 53:112–119. 10.1016/j.soilbio.2012.05.008

[PLV030C12] Burns JH Strauss SY 2011 More closely related species are more ecologically similar in an experimental test. Proceedings of the National Academy of Sciences of the USA 108:5302–5307. 10.1073/pnas.1013003108 21402914 PMC3069184

[PLV030C13] Comas LH Eissenstat DM 2009 Patterns in root trait variation among 25 co-existing North American forest species. New Phytologist 182:919–928. 10.1111/j.1469-8137.2009.02799.x 19383105

[PLV030C14] Compant S Clément C Sessitsch A 2010 Plant growth-promoting bacteria in the rhizo- and endosphere of plants: their role, colonization, mechanisms involved and prospects for utilization. Soil Biology and Biochemistry 42:669–678. 10.1016/j.soilbio.2009.11.024

[PLV030C15] Desdevises Y Legendre P Azouzi L Morand S 2003 Quantifying phylogenetically structured environmental variation. Evolution 57:2647–2652. 10.1111/j.0014-3820.2003.tb01508.x 14686540

[PLV030C16] Diniz-Filho JAF de Sant’Ana CER Bini LM 1998 An eigenvector method for estimating phylogenetic inertia. Evolution 52:1247–1262. 10.2307/2411294 28565378

[PLV030C17] Diniz-Filho JAF Bini LM Rangel TF Morales-Castilla I Olalla-Tárraga MA Rodríguez MA Hawkins BA 2012 On the selection of phylogenetic eigenvectors for ecological analyses. Ecography 35:239–249. 10.1111/j.1600-0587.2011.06949.x

[PLV030C18] Edgar RC 2004a MUSCLE: a multiple sequence alignment method with reduced time and space complexity. BMC Bioinformatics 5:1–19. 10.1186/1471-2105-5-113 15318951 PMC517706

[PLV030C19] Edgar RC 2004b MUSCLE: multiple sequence alignment with high accuracy and high throughput. Nucleic Acids Research 32:1792–1797. 10.1093/nar/gkh340 15034147 PMC390337

[PLV030C20] Ehrenfeld JG Ravit B Elgersma K 2005 Feedback in the plant-soil system. Annual Review of Environment and Resources 30:75–115. 10.1146/annurev.energy.30.050504.144212

[PLV030C21] Ettema CH Wardle DA 2002 Spatial soil ecology. Trends in Ecology and Evolution 17:177–183. 10.1016/S0169-5347(02)02496-5

[PLV030C22] Faith DP Minchin PR Belbin L 1987 Compositional dissimilarity as a robust measure of ecological distance. Vegetatio 69:57–68. 10.1007/BF00038687

[PLV030C23] Fierer N Jackson RB 2006 The diversity and biogeography of soil bacterial communities. Proceedings of the National Academy of Sciences of the USA 103:626–631. 10.1073/pnas.0507535103 16407148 PMC1334650

[PLV030C24] Fierer N Ladau J 2012 Predicting microbial distributions in space and time. Nature Methods 9:549–551. 10.1038/nmeth.2041 22669651

[PLV030C25] Fierer N Leff JW Adams BJ Nielsen UN Bates ST Lauber CL Owens S Gilbert JA Wall DH Caporaso JG 2012 Cross-biome metagenomic analyses of soil microbial communities and their functional attributes. Proceedings of the National Academy of Sciences of the USA 109:21390–21395. 10.1073/pnas.1215210110 23236140 PMC3535587

[PLV030C26] Fox J Monette G 1992 Generalized collinearity diagnostics. Journal of the American Statistical Association 87:178–183. 10.1080/01621459.1992.10475190

[PLV030C27] Gonçalves-Souza T Diniz-Filho JAF Romero GQ 2014 Disentangling the phylogenetic and ecological components of spider phenotypic variation. PLoS ONE 9:e89314 10.1371/journal.pone.0089314 24651264 PMC3942061

[PLV030C28] Hardoim PR van Overbeek LS van Elsas JD 2008 Properties of bacterial endophytes and their proposed role in plant growth. Trends in Microbiology 16:463–471. 10.1016/j.tim.2008.07.008 18789693

[PLV030C29] Innes L Hobbs PJ Bardgett RD 2004 The impacts of individual plant species on rhizosphere microbial communities in soils of different fertility. Biology and Fertility of Soils 40:7–13. 10.1007/s00374-004-0748-0

[PLV030C30] Jacquemyn H Merckx V Brys R Tyteca D Cammue BPA Honnay O Lievens B 2011 Analysis of network architecture reveals phylogenetic constraints on mycorrhizal specificity in the genus *Orchis* (Orchidaceae). New Phytologist 192:518–528. 10.1111/j.1469-8137.2011.03796.x 21668874

[PLV030C31] Kluber LA Carrino-Kyker SR Coyle KP DeForest JL Hewins CR Shaw AN Smemo KA Burke DJ 2012 Mycorrhizal response to experimental pH and P manipulation in acidic hardwood forests. PLoS ONE 7:e48946 10.1371/journal.pone.0048946 23145035 PMC3493595

[PLV030C32] Kulmatiski A Beard KH Stevens JR Cobbold SM 2008 Plant-soil feedbacks: a meta-analytical review. Ecology Letters 11:980–992. 10.1111/j.1461-0248.2008.01209.x 18522641

[PLV030C33] Lauber CL Hamady M Knight R Fierer N 2009 Pyrosequencing-based assessment of soil pH as a predictor of soil bacterial community structure at the continental scale. Applied and Environmental Microbiology 75:5111–5120. 10.1128/AEM.00335-09 19502440 PMC2725504

[PLV030C34] Leake JR Donnelly DP Boddy L 2002 Interactions between ecto-mycorrhizal and saprotrophic fungi. In: Heijden MGA Sanders IR, eds. Mycorrhizal ecology. Berlin: Springer, 345–371.

[PLV030C35] Liu X Liang M Etienne RS Wang Y Staehelin C Yu S 2012 Experimental evidence for a phylogenetic Janzen-Connell effect in a subtropical forest. Ecology Letters 15:111–118. 10.1111/j.1461-0248.2011.01715.x 22082078

[PLV030C36] Long SR 2001 Genes and signals in the Rhizobium-legume symbiosis. Plant Physiology 125:69–72. 10.1104/pp.125.1.69 11154299 PMC1539328

[PLV030C37] Lugo MA Reinhart KO Menoyo E Crespo EM Urcelay C 2015 Plant functional traits and phylogenetic relatedness explain variation in associations with root fungal endophytes in an extreme arid environment. Mycorrhiza 25:85–95. 10.1007/s00572-014-0592-5 24997550

[PLV030C38] Manjunath A Habte M 1991 Root morphological-characteristics of host species having distinct mycorrhizal dependency. Canadian Journal of Botany-Revue Canadienne De Botanique 69:671–676.

[PLV030C39] Martos F Munoz F Pailler T Kottke I Gonneau C Selosse M-A 2012 The role of epiphytism in architecture and evolutionary constraint within mycorrhizal networks of tropical orchids. Molecular Ecology 21:5098–5109. 10.1111/j.1365-294X.2012.05692.x 22765763

[PLV030C40] McCune B Grace JB 2002 Analysis of ecological communities. MjM Software Design, Gleneden Beach, OR, USA, 304 pp.

[PLV030C41] Mehrabi Z Tuck SL 2015 Relatedness is a poor predictor of negative plant-soil feedbacks. New Phytologist 205:1071–1075. 10.1111/nph.13238 25557183 PMC4303931

[PLV030C42] O'Brien RM 2007 A caution regarding rules of thumb for variance inflation factors. Quality and Quantity 41:673–690. 10.1007/s11135-006-9018-6

[PLV030C43] Oksanen J Blanchet FG Kindt R Legendre P O'Hara RB Simpson GL Solymos P Stevens MHH Wagner H 2010 Vegan: community ecology package. R package version 1.17-2 ed.

[PLV030C44] O'Malley MA 2007 The nineteenth century roots of ‘everything is everywhere’. Nature Reviews Microbiology 5:647–651. 10.1038/nrmicro1711 17603517

[PLV030C45] Peres-Neto PR Legendre P Dray S Borcard D 2006 Variation partitioning of species data matrices: estimation and comparison of fractions. Ecology 87:2614–2625. 10.1890/0012-9658(2006)87[2614:VPOSDM]2.0.CO;2 17089669

[PLV030C46] Pilloni G Granitsiotis MS Engel M Lueders T 2012 Testing the limits of 454 pyrotag sequencing: reproducibility, quantitative assessment and comparison to T-RFLP fingerprinting of aquifer microbes. PLoS ONE 7:e40467 10.1371/journal.pone.0040467 22808168 PMC3395703

[PLV030C47] R Development Core Team. 2008 R: a language and environment for statistical computing, 2.8.1 ed Vienna, Austria: R Foundation for Statistical Computing.

[PLV030C48] Reinhart KO Anacker BL 2014 More closely related plants have more distinct mycorrhizal communities. AoB PLANTS 6:plu051; doi:10.1093/aobpla/plu051 10.1093/aobpla/plu051 25165062 PMC4172195

[PLV030C49] Reinhart KO Wilson GWT Rinella MJ 2012 Predicting plant responses to mycorrhizae: integrating evolutionary history and plant traits. Ecology Letters 15:689–695. 10.1111/j.1461-0248.2012.01786.x 22507627

[PLV030C50] Revell LJ 2012 phytools: an R package for phylogenetic comparative biology (and other things). Methods in Ecology and Evolution 3:217–223. 10.1111/j.2041-210X.2011.00169.x

[PLV030C51] Rout ME Callaway RM 2012 Interactions between exotic invasive plants and soil microbes in the rhizosphere suggest that ‘everything is not everywhere’. Annals of Botany 110:213–222. 10.1093/aob/mcs061 22451600 PMC3394644

[PLV030C52] Rudrappa T Czymmek KJ Pare PW Bais HP 2008 Root-secreted malic acid recruits beneficial soil bacteria. Plant Physiology 148:1547–1556. 10.1104/pp.108.127613 18820082 PMC2577262

[PLV030C53] Sanderson MJ 2003 r8s: inferring absolute rates of molecular evolution and divergence times in the absence of a molecular clock. Bioinformatics 19:301–302. 10.1093/bioinformatics/19.2.301 12538260

[PLV030C54] Singh BK Millard P Whiteley AS Murrell JC 2004 Unravelling rhizosphere–microbial interactions: opportunities and limitations. Trends in Microbiology 12:386–393. 10.1016/j.tim.2004.06.008 15276615

[PLV030C55] Stevens PF 2009 Angiosperm phylogeny website. Version 9 ed.

[PLV030C56] Tamura K Peterson D Peterson N Stecher G Nei M Kumar S 2011 MEGA5: molecular evolutionary genetics analysis using maximum likelihood, evolutionary distance, and maximum parsimony methods. Molecular Biology and Evolution 28:2731–2739. 10.1093/molbev/msr121 21546353 PMC3203626

[PLV030C57] Taylor DL Hollingsworth TN McFarland JW Lennon NJ Nusbaum C Ruess RW 2014 A first comprehensive census of fungi in soil reveals both hyperdiversity and fine-scale niche partitioning. Ecological Monographs 84:3–20. 10.1890/12-1693.1

[PLV030C58] Ushio M Wagai R Balser TC Kitayama K 2008 Variations in the soil microbial community composition of a tropical montane forest ecosystem: does tree species matter? Soil Biology and Biochemistry 40:2699–2702. 10.1016/j.soilbio.2008.06.023

[PLV030C59] Valverde-Barrantes OJ Smemo KA Blackwood CB 2014 Fine root morphology is phylogenetically structured, but nitrogen is related to the plant economics spectrum in temperate trees. Functional Ecology; 10.1111/1365-2435.12384.

[PLV030C60] Wikström N Savolainen V Chase MW 2001 Evolution of the angiosperms: calibrating the family tree. Proceedings of the Royal Society B: Biological Sciences 268:2211–2220. 10.1098/rspb.2001.1782 PMC1088868 11674868

[PLV030C61] Zwickl DJ 2006 Genetic algorithm approaches for the phylogenetic analysis of large biological sequence datasets under the maximum likelihood criterion. PhD Dissertation, The University of Texas, Austin.

