# Supplementary material for: Soil microbial community variation correlates most strongly with plant species identity, followed by soil chemistry, spatial location and plant genus
Source: AoB Plants. 2015 Mar 27;7:plv030. doi: 10.1093/aobpla/plv030 (PMC4417136; doi:10.1093/aobpla/plv030)
Supplement: Additional Information [file supp_7_plv030_index.html]

Soil microbial community variation correlates most strongly with plant species identity, followed by soil chemistry, spatial location and plant genus — Additional Information 

# Soil microbial community variation correlates most strongly with plant species identity, followed by soil chemistry, spatial location and plant genus

## Additional Information

Additional Information

**Files in this Data Supplement:**

- Supplementary Data - Doc file
