# Supplementary material for: Soil microbial community variation correlates most strongly with plant species identity, followed by soil chemistry, spatial location and plant genus
Source: AoB Plants. 2015 Mar 27;7:plv030. doi: 10.1093/aobpla/plv030 (PMC4417136; doi:10.1093/aobpla/plv030)
Supplement: Additional Information [file supp_plv030_plv030supp.doc]

**Supporting Information**

**Table S1** Angiosperm species sampled to examine rhizosphere soil microbial communities at Bodega Bay, CA, USA.

| Family | Species | Habitat | Life form | Known AM association | *mat*K | ITS | *trn*L-*trn*F |
| --- | --- | --- | --- | --- | --- | --- | --- |
| Asteraceae | *Cirsium occidentale* | Dune | Biennial Perennial | AM confirmed in other *Cirsium* sp. (Wang and Qiu 2006) | KC969514 | AF443702 | KC969599 |
| Asteraceae | *Cirsium quercetorum* | Grassland | Biennial Perennial | AM confirmed in other *Cirsium* sp. (Wang and Qui 2006) | KC969516 | AF443706 | KC969601 |
| Rosaceae | *Fragaria chiloensis* | Dune | Perennial | AM (Wang and Qui 2006) | NA | AF163514 | FJ422293 |
| Rosaceae | *Fragaria vesca* | Wetland | Perennial | AM in some studies (Wang and Qui 2006) | HM850686 | AF163510 | AF348545 |
| Polemoniaceae | *Gilia capitata* ssp. *chamissonis* | Dune | Annual  Perennial | AM | L34182 | EU339740 | EU348391 |
| Polemoniaceae | *Gilia millefoliata* | Dune | Annual | AM | NA | AF202946 | AF011441 |
| Plantaginaceae | *Plantago erecta* | Grassland | Annual | AM confirmed in other *Plantago* sp. (Wang and Qui 2006) | NA | AY101909 | AY101962 |
| Plantaginaceae | *Plantago subnuda* | Grassland (disturbed) | Perennial | AM confirmed in other *Plantago* sp. (Wang and Qui 2006) | NA | HQ593836 | HQ593821 |
| Polygonaceae | *Rumex crassus*^a^ | Dune | Perennial | AM mostly absent in this genus (Wang and Qui 2006) | HQ593423 | JQ288757 | JQ041851 |
| Polygonaceae | *Rumex occidentalis* | Wetland | Perennial | AM mostly absent in this genus (Wang and Qui 2006) | KC475697 | NA | NA |
| Apiaceae | *Sanicula arctopoides* | Grassland (rocky) | Perennial | AM confirmed in other *Sanicula* sp. (Wang and Qui 2006) | NA | EU070743 | NA |
| Apiaceae | *Sanicula crassicaulis* | Woodland | Perennial | AM confirmed in other *Sanicula* sp. (Wang and Qui 2006) | NA | AF031988 | NA |
| Fabaceae | *Trifolium fucatum* | Grassland (rocky) | Annual | AM associations recorded for many *Trifolium* sp. (Wang and Qui 2006) | NA | DQ312054 | DQ311792 |
| Fabaceae | *Trifolium gracilentum* | Grassland | Annual | AM associations recorded for many *Trifolium* sp. (Wang and Qui 2006) | AF522123 | DQ312060 | DQ311798 |

^a^*Rumex crispus* was used as a substitute for phylogenetic analysis, because genetic data was not available in genBank for *R. crassus*. GenBank accession numbers for each taxa are reported for sequences used to estimate the phylogeny.

Fig. S1. Soils were sampled from Bodega Bay Marine Reserve, California, USA. cc = *Cirsium occidentale,* cq *= C. quercetorum*, fc = *Fragaria chiloensis*, fv = *F. vesca*, gc = *Gilia capitata* ssp. *chamissonis*, gm = *G. millefoliata*, pe = *Plantago erecta*, ps = *P. subnuda*, rc = *Rumex crassus*, ro = *R. occidentalis*, sa = *Sanicula arctopoides*, sc = *S. crassicaulis*, tf = *Trifolium fucatum* and tg = *T. gracilentum*

**Terminal Restriction Fragment Length Polymorphism, Supplemental Methods Details**

DNA was extracted from each soil subsample using a CTAB bead-beating protocol and purified using a phenol-chloroform extraction (Bur*ke et a*l. 2012). For each extraction, we used approximately 500-mg of field soil and placed soil into a bead beating tube containing 500-mg of sterile glass beads (300-mg of 400 μM glass beads [VWR,West Chester, PA, USA], 200-mg 1 mm glass beads [Chemglass, Vineland, NJ, USA]) and 750 mL of 2% CTAB (cetyltrimethyl-ammonium bromide). Samples were then beaten for 40 seconds in a Precellys homogenizer (Bertin Technologies, Montigny-le-Bretonneux, France) at 6500 rpm. Samples were purified using phenol-chloroform extraction followed by precipitation with 20% polyethylene glycol 8000 in 2.5 M NaCl. DNA was suspended in 100-μl Tris EDTA buffer and stored at -20ºC until use (Burke et al. 2012).

To isolate bacteria and fungal-specific DNA regions, we conducted PCR with primers specific to those taxonomic groups. We targeted the 16S rRNA gene for bacteria using primers 338f and 926r (Muyz*er et a*l. 1993, Muyz*er et a*l. 1995) following conditions described by Burke et al. (2008). For fungi, we targeted the ITS2 region of the rRNA gene using primers 58A2F and NLB4 (Martin and Rygiewicz 2005) following conditions described in Burke et al. (2005) except the extension step was increased to 90 seconds. The ITS2 primers preferentially amplify non-arbuscular mycorrhizal fungi (Burke, per. obs.; Tayl*or et a*l. 2014), and were chosen for their generality—to create a data set with similar breadth to the bacterial data set. PCR was conducted using primers fluorescently labeled with either 6FAM (6-carboxyfluorescein) or HEX (4, 7, 20, 40, 50, 70 -hexachloro-6-carboxyfluorescein) and was carried out in 50-μl reaction volumes using 1-μl of purified DNA and 1 or 2 units of Taq DNA polymerase (Promega, Madison, WI) for bacteria and fungi respectively. Products were confirmed using agarose gel electrophoresis with positive and negative controls.

To generate DNA fragments, we fragmented the PCR product with restriction enzymes. Restriction digests were conducted using *Msp*I for bacteria and *Hae*III for fungi, using protocols previously described (Bur*ke et a*l. 2005, Bur*ke et a*l. 2008) with incubation for 4 hours, followed by 15 min denaturing at 65°C. Terminal restriction fragment length polymorphisms (TRFLP) were completed through the Life Sciences Core Laboratories Center (Cornell University) on an 3730xl DNA Analyzer using the GS600 LIZ size standard and Peak ScannerTM Software (version 1.0, Applied Biosystems 2006). Only peaks that accounted for > 1% of the relative peak area (i.e. major TRFs; Burke *et al*., 2008) were included in the analysis. We have found that peaks less than 1% of total profile area are generally not repeatable between replicate samples, and although excluding these peaks may provide a more conservative estimate of microbial diversity, it reduces the chance that non-specific TRFs will be included in our analysis (Burke *et al*. 2008).

The subsamples that did not amplify with PCR were not included in subsequent analysis (see Results). The bacteria data set contained 157 samples that could be amplified, out of a possible 168 (14 species × 6 sampling locations × 2 subsamples per soil core) with 179 bacterial OTUs. The fungal data set contained 146 samples (out of a possible 168) that could be amplified with 198 OTUs.

To summarize the soil community data, we assigned TRFLP peaks to OTUs with a ± 0.25 bp bin width (Bur*ke et a*l. 2008), resulting in community matrices with bacteria and fungal OTUs for each soil extraction. With the TRFLP approach used here, TRF size between replicate profiles are generally less than 0.25 bp (Bur*ke et a*l. 2008). We present TRFLP data from the reverse primer (NLB4) for fungi and the forward primer (338F) for bacteria because these labeled primers generated the largest number of OTUs (i.e. TRFs) for both bacterial and fungal data sets. Singleton OTUs, those appearing only once in the data set, can arise from experimental error and were removed following standard procedure (Klub*er et a*l. 2012). The resulting subsample matrices were then averaged. The resulting data sets are matrices of community data for each soil sample, with relative abundances of each bacterial or fungal OTU.

**Variance Partitioning analysis**

Variance partitioning analysis was used to qualitatively describe sources of variance in the data set. Note that there has been controversy over the application of these methods, and simulation studies have found effect sizes to be biased (Gilbert and Bennett 2010). However, we use these analyses as a data-exploration tool. For each of group (bacteria, fungi) and for each ordination axis (axes 1 – 4), we fit seven multiple regression models:

m1: soil chemistry (8 variables; described below)

m2: phylogeny (1-5 phylogenetic eigenvectors)

m3: spatial location (2 eigenvectors; described below)

m4: soil chemistry + spatial location

m5: soil chemistry + phylogeny

m6: spatial location + phylogeny

m7: soil chemistry + spatial location + phylogeny

For the phylogeny model, the phylogeny was represented in linear terms by first decomposing the phylogeny into a set of principal coordinates (PCs) using the *PVRdecomp* function of the PVR package, regressing each PC against each ordination axis, retaining those that were significantly related at alpha 0.05. For the soil chemistry model, we included CEC, Ca, N, P, K, Na, Mg, and pH; OM was not included, because it was highly correlated with CEC (r = 0.72). For the spatial location model, we decomposed the spatial coordinates into two eigenvectors using a principal coordinates analysis via the *pco* function of the ecodist package.

Next, we derived the constituent components via subtraction of the R^2^ values from the seven regression models (Desdevis*es et a*l. 2003). For example, the soil chemistry only R^2^ component = m7 R^2^ – m6 R^2^; it represents the relationship of soil community structure and soil chemistry, after taking into account spatial location and plant phylogeny.

**Fig S2.** Variance partitioning analysis on the bacterial and fungal data sets. Predictors included spatial location, soil chemistry, plant phylogeny, and all possible combinations of these predictors.

**Table S2.** Linear PVR models were used to test the effects of plant species, soil chemistry, spatial location, and plant genus on soil microbial community composition.

|  |  | Microbial community structure ordination axes | | | | | | | |
| --- | --- | --- | --- | --- | --- | --- | --- | --- | --- |
|  | Predictor | MDS1 | | MDS2 | | MDS3 | | MDS4 | |
|  |  | Adj R^2^ | P-value | Adj R^2^ | P-value | Adj R^2^ | P-value | Adj R^2^ | P-value |
| Bacteria | Plant species | <0.01 | 0.42 | **0.15** | **0.02** | **0.15** | **0.02** | **0.38** | **<0.001** |
|  | Soil chemistry | <0.01 | 0.77 | **0.19** | **<0.001** | **0.16** | **<0.01** | **0.11** | **0.05** |
|  | Spatial location | <0.01 | 0.14 | <0.01 | 0.90 | **0.19** | **<0.01** | **0.16** | **< 0.001** |
|  | Genus | <0.01 | 0.69 | 0.07 | 0.07 | **0.12** | **0.02** | 0.05 | 0.12 |
| Fungi | Plant species | **0.56** | **< 0.001** | **0.31** | **< 0.001** | **0.20** | **<0.01** | 0.03 | 0.31 |
|  | Soil chemistry | **0.45** | **< 0.001** | **0.27** | **< 0.001** | <0.01 | 0.17 | 0.03 | 0.06 |
|  | Spatial location | **0.43** | **< 0.001** | <0.01 | 0.93 | **0.09** | **< 0.001** | **0.10** | **0.02** |
|  | Genus | **0.16** | **<0.01** | **0.30** | **< 0.001** | <0.01 | 0.35 | <0.01 | 0.43 |

Model selection was conducted by retaining statistically significant phylogenetic eigenvectors (P < 0.05) for the combined data set (see text for details). Significant results (P < 0.05) are highlighted in bold.

**References**

Burke DJ, Dunham SM, Kretzer AM. 2008. Molecular analysis of bacterial communities associated with the roots of Douglas fir (Pseudotsuga menziesii) colonized by different ectomycorrhizal fungi. *Fems Microbiology Ecology,* 65: 299-309.

Burke DJ, Martin KJ, Rygiewicz PT, Topa MA. 2005. Ectomycorrhizal fungi identification in single and pooled root samples: terminal restriction fragment length polymorphism (TRFLP) and morphotyping compared. *Soil Biology & Biochemistry,* 37: 1683-1694.

Burke DJ, Smemo KA, Lopez-Gutierrez JC, DeForest JL. 2012. Soil fungi influence the distribution of microbial functional groups that mediate forest greenhouse gas emissions. *Soil Biology & Biochemistry,* 53: 112-119.

Desdevises Y, Legendre P, Azouzi L, Morand S. 2003. Quantifying phylogenetically structured environmental variation. *Evolution,* 57: 2647-2652.

Gilbert B, Bennett JR. 2010. Partitioning variation in ecological communities: do the numbers add up? *Journal of Applied Ecology,* 47: 1071-1082.

Kluber LA, Carrino-Kyker SR, Coyle KP, DeForest JL, Hewins CR, Shaw AN, Smemo KA, Burke DJ. 2012. Mycorrhizal Response to Experimental pH and P Manipulation in Acidic Hardwood Forests. *Plos One,* 7: 1-10.

Martin KJ, Rygiewicz PT. 2005. Fungal-specific PCR primers developed for analysis of the ITS region of environmental DNA extracts. *Bmc Microbiology,* 5.

Muyzer G, Dewaal EC, Uitterlinden AG. 1993. Profiling of complex microbial-populations by denaturing gradient gel-electrophoresis analysis of polymerase chain reaction-amplified genes-coding for 16S ribosomal-RNA. *Applied and Environmental Microbiology,* 59: 695-700.

Muyzer G, Teske A, Wirsen CO, Jannasch HW. 1995. Phylogenetic-relationships of Thiomicrospira species and their identification in deep-sea hydrothermal vent samples by denaturing gradient gel-electophoresis of 16S RDNA fragments. *Archives of Microbiology,* 164: 165-172.

Taylor DL, Hollingsworth TN, McFarland JW, Lennon NJ, Nusbaum C, Ruess RW. 2014. A first comprehensive census of fungi in soil reveals both hyperdiversity and fine-scale niche partitioning. *Ecological Monographs,* 84: 3-20.

Wang B, Qiu YL. 2006. Phylogenetic distribution and evolution of mycorrhizas in land plants. *Mycorrhiza,* 16: 299-363.
